# Adopting an heterologous prime-boost strategy in COVID-19 vaccination: the need for locally generated evidence in Africa

**DOI:** 10.11604/pamj.2022.41.148.31620

**Published:** 2022-02-18

**Authors:** Mary Aigbiremo Oboh, Semeeh Omoleke, Kolawole Salami

**Affiliations:** 1 Medical Research Council Unit, The Gambia at London School of Hygiene and Tropical Medicine, Fajara, Banjul, The Gambia,; 2 Malaria Genomic Epidemiology Network, Nigeria, Nigerian Institute of Medical Research, Lagos, Nigeria,; 3 Field Presence, World Health Organization, Adamawa State Field Office, Adamawa, Nigeria,; 4 Research and Development Blueprint Unit, World Health Organization, Geneva, Switzerland

**Keywords:** COVID-19-heterologous vaccine, prime-boost dose, reactogenicity, safety, immunogenicity

## Abstract

The reduction in the severity and prevalence of COVID-19 has been largely due to the rapid development and deployment of COVID-19 vaccines. Consequently, WHO, in partnership with the Coalition for Epidemic Preparedness Innovation, GAVI, the Vaccine Alliance, set up the COVID-19 Vaccines Global Access (COVAX) Initiative. The goal of this initiative is to prevent discrimination between high and low-income/middle-income countries and ensure equitable vaccine distribution. The first COVID-19 vaccine sent to most countries in the region through the COVAX initiative was the Oxford AstraZeneca (ChAdOx1 nCoV-19) vaccine. Due to the reduced protection against variants of concern, safety issues, and supply challenges of the AstraZeneca vaccine in some countries, heterologous booster dose with alternative vaccines for individuals who have received a prime dose of AstraZeneca. Moreover, vaccine mixing (heterologous vaccination) due to its superior immunogenicity and enhanced protection is being recommended even for individuals who are yet to be vaccinated. However, it is important that prior adoption, empirical data on immunogenicity, safety, and reactogenicity be locally generated in populations where such heterologous vaccine is to be implemented. Regrettably, such data from our search in all clinical trial databases is not ongoing in Africa as at the time of writing this manuscript. Therefore, this treatise advocates an experimental arm to generate such robust evidence. This will provide empirical evidence to guide this innovative approach aimed at ensuring equity and access to COVID-19 vaccines in LMICs, particularly countries within the African region.

## Commentary

The detection of the novel coronavirus disease (COVID-19) in China; in December 2019 and its subsequent declaration as a public health emergency of international concern on 30 January 2020 has significantly impacted global health systems, economies, and travel. The African region has recorded a relatively modest infection rate of infection. As of 2 September 2021, Africa has an estimated 7 849 859 cases and 198 132 mortality, representing 0.04% and 0.04% of the global estimates [1]. In the absence of effective treatment, the rapid development of vaccines using innovative platforms offers a glimmer of hope. The World Health Organisation (WHO) has approved some of the vaccines for emergency use [2]. With the tremendous success of COVID-19 vaccine development, it becomes imperative to set a robust strategic plan for equitable distribution, affordability, and accessibility to every individual regardless of political, racial or cultural background.

Consequently, WHO, in partnership with the Coalition for Epidemic Preparedness Innovation (CEPI), GAVI, the Vaccine Alliance, set up the COVID-19 Vaccines Global Access (COVAX) Initiative, which aims to accelerate the manufacture and equitable access of COVID-19 vaccines to all countries irrespective of their economic status. The COVAX initiative proposed to deliver approximately 2 billion doses of COVID-19 vaccines by the end of 2021 to frontline workers and individuals who are > 65years with or without co-morbidities. At conception, the ultimate goal of this distribution initiative is to prevent discrimination between high and low income/middle income countries (LMICs), and in turn, ensure equitable vaccine distribution. COVAX and the World Bank hopes to accelerate COVID-19 vaccine supply for developing countries through a new financing mechanism that builds on GAVI´s newly designed arthrogryposis multiplex congenita (AMC) cost-sharing arrangement. Therefore, countries within the African region can capitalise on this distribution plan to access the COVID-19 vaccine and effect strategic distribution across the continent and nationwide. However, hoarding and vaccine nationalism by wealthier nations have slowed the COVAX initiatives' implementation and hindered the realization of its ambitious drive. The first COVID-19 vaccine sent to most countries in the region through the COVAX initiative was the Oxford AstraZeneca (ChAdOx1 nCoV-19) vaccine. However, the supply dwindled due to a decline in production capacity caused by an upsurge in COVID-19 cases in India, where the manufacturer (Serum Institute of India, Pvt) of AstraZeneca vaccine is located.

As of 9^th^ August 2021, reports on COVID-19 vaccination coverage have not been encouraging in countries within the African region. Only 1.56% and 3.71% of the population have received two (2) and one (1) doses of the various COVID-19 vaccines. Only two countries have vaccinated more than 50% of its population (Seychelles- 95% and Mauritius - 75%), and six have vaccinated almost a quarter of its population (Morocco, South Africa, Djibouti, Equatorial Guinea, Eswatini, and Cape Verde), while some (Burundi, Eritrea and Sierra Leone) are yet to provide any vaccination indices [2]. The setbacks in access and coverage would impact on transmission negatively and spread of variants SARS-CoV-2 strains by each country and the continent in general, given the suboptimal adherence to public health and social measures.

As a result of the changing recommendations on the use of the AstraZeneca vaccine regarding its reduced protection against variants of concern in some countries [3], high income countries are now recommending that individuals previously primed with the AstraZeneca vaccine should receive an alternative vaccine as their second (boost) dose. In addition, alternative prime-boost vaccination has been found to provoke superior immunogenicity and enhanced protection against SARS-CoV-2 [4] as well as against Ebola [5]. Messenger Ribonucleic Acid (mRNA) vaccine has been suggested as a reliable boost dose [6]. Hence, a heterologous prime-boost COVID-19 vaccine scenario is gaining prominence. Consequently, with African countries getting various COVID-19 vaccines based on different technology platforms, secured through different initiatives, to ensure as much coverage as possible, (Table 1, Table 1 (suite), Table 1 (suite 1), Table 1 (suite 2)) it is expected and indeed planned by some of these countries that most people who received an incomplete AstraZeneca dose (a single dose of the vaccine), or have not received any vaccination, will receive a second (for complete vaccination) or full dose course of another vaccine. Theoretically, this serves as the booster vaccine.

**Table 1:** different COVID-19 in African countries and percentage of population vaccinated

Countries	COVID-19 vaccines	Number of vaccine supplied	Number of vaccine administered	Percentage of population fully vaccinated
South Africa	BNT162b2, Johnson and Johnson	14 408 830	7 567 757	24.3
Morocco	AstraZeneca, Sinopharm, Johnson and Johnson	27 109 400	24 751,744	0.57
Egypt	AstraZeneca, Sinopharm,	7 323 200	5 582 316	1.76
Nigeria	AstraZeneca, Moderna	7 940 000	3 938 945	0.68
Tunisia	AstraZeneca, Sinopharm, Sputnik V, BNT162b2, Moderna	3 575 890	2 663 438	8.17
Zimbabwe	Sinopharm, Sputnik V	4 900 000	2 540 555	5.59
Algeria	AstraZeneca Sinopharm Sputnik V,	3 673 200	2 500 000	0
Ethiopia	AstraZeneca Sinopharm, Johnson and Johnson	3 344 800	2 277 783	0.04
Kenya	AstraZeneca	1 706 100	1 734 013	1.24
Angola	AstraZeneca, Sinopharm, Sputnik V, BNT162b2	1 525 240	1 674 157	2.14
Senegal	AstraZeneca Sinopharm Johnson and Johnson	1 476 200	1 388 153	2.92
Ghana	AstraZeneca	1 331 000	1 271 393	1.31
Mauritius	AstraZeneca Sinopharm Sputnik V	1 974 000	1 252 974	43.21
Uganda	AstraZeneca Sinovac	1 725 28	1 152 874	0

**Table 1(suite):** different COVID-19 in African countries and percentage of population vaccinated

Côte d'Ivoire	AstraZeneca Sinopharm	1 123 420	976 545	0.44
Guinea	AstraZeneca Sinopharm Sputnik V Sinovac	985 460	904 845	2.59
Rwanda	AstraZeneca BNT162b2 Moderna	1 280 320	868 972	3.02
Sudan	AstraZeneca Sinopharm	1 050 000	810 560	0.42
Libya	AstraZeneca Sinopharm Sputnik V	580 190	712 213	0
Malawi	AstraZeneca Johnson and Johnson	876 390	597 862	0.74
Togo	AstraZeneca BNT162b2 Sinovac	576 620	474 776	1.85
Zambia	AstraZeneca Sinopharm Johnson and Johnson	620 800	444 574	0.86
Niger	AstraZeneca Sinopharm Johnson and Johnson	931 400	401 133	0.24
Botswana	AstraZeneca Sinovac	492 400	365 137	5.31
Cameroon	AstraZeneca Sinopharm Johnson and Johnson	1 045 900	331 875	0.2
Mozambique	AstraZeneca Sinopharm Johnson and Johnson	1 704 400	318 004	0.25
Equatorial Guinea	Sinopharm	600 000	308 858	9.17

**Table 1(suite 1):** different COVID-19 in African countries and percentage of population vaccinated

Somalia	AstraZeneca Sinopharm	608 000	249 790	0.55
Mauritania	AstraZeneca Sinopharm, BNT162b2 Johnson and Johnson	839 600	224 000	0.41
Namibia	AstraZeneca Sinopharm	197 200	218 453	2.02
Mali	AstraZeneca	396 000	206 562	0.28
Congo(Republic)	Sinopharm Sputnik V	172 000	198 698	1.2
Madagascar	AstraZeneca Johnson and Johnson	552 750	197 001	0
Cabo Verde	AstraZeneca Sinopharm, BNT162b2	211 050	183 620	3.61
Sierra Leone	AstraZeneca Sinopharm	324 125	163 085	0.3
Sechelles	AstraZeneca Sinopharm Sputnik V	190 000	139 625	68.14
Comoros	AstraZeneca Sinopharm	112 000	134 020	4.79
Gabon	Sinopharm	300 000	103 820	1.93
Liberia	AstraZeneca Johnson and Johnson	700 800	95 423	0.18
Central African Republic	AstraZeneca Johnson and Johnson	382 400	95 282	0.21
Democratic Republic of Congo	AstraZeneca	351 000	86 170	0.01

**Table 1(suite 2):** different COVID-19 in African countries and percentage of population vaccinated

Benin	AstraZeneca Johnson and Johnson Sinovac	649 400	70 323	0.18
Eswatini	AstraZeneca Johnson and Johnson	512 000	65 667	2.28
South Sudan	AstraZeneca	132 000	56 989	0.04
Djibouti	AstraZeneca Johnson and Johnson Sinovac	475 200	50 509	1.9
Sao Tome and Principe	AstraZeneca	53 850	43 960	5.94
Gambia	AstraZeneca Johnson and Johnson	202 200	43 557	0.51
Lesotho	AstraZeneca Sinopharm Johnson and Johnson	510 000	38 320	0.38
Chad	AstraZeneca Sinopharm	300 620	36 173	0.06
Burkina Faso	AstraZeneca Johnson and Johnson	266 400	35 402	0
Guinea-Bissau	AstraZeneca	64 800	25 872	0.09
Tanzania	Johnson and Johnson	1 058 400	0	0
Sahrawi Republic	AstraZeneca	20 000	0	0

Although, some studies which evaluated heterologous prime-boost COVID-19 vaccine of different platforms have reported higher frequency of spike-specific CD4+, CD8+ T-cells [4], strong and high titers of neutralising antibodies against B1.1.7 and B.1.351 strains [6,7]. Reactogenicity after heterologous boost was reported as vaccine unrelated in one study (flu-like illness, headache, asthenia) [8], while another [9] attributed increased systemic reactogenicity after boost dose to heterologous manner. The Shaw [9] study which was conducted in individuals >50 years and older in the UK, suggested that reactogenicity might be higher if younger individuals were administered heterologous prime-boost doses. Although the reactogenicity observed is short-term, such trial needs to be replicated in various populations with the different vaccine combinations intended for implementation. All the aforementioned studies were conducted in Germany, Russia, China, and the UK. None has been conducted in any African countries that have or are about to commence implementing heterologous boost dose. A thorough search of clinical trial registries (clinicaltrial.gov, ICTRP, ISRCTN, PACTR, etc.) for COVID-19 heterologous vaccine efficacy regrettably did not return any hit from any African country. The importance of carrying out such a study in different African settings/countries cannot be overemphasised, especially as the emergence of new variants is quite rapid.

## Conclusion

Globally, heterologous prime-boost vaccines offer enhanced protection in comparison to homologous vaccine, however, it is important that prior adoption, safety and reactogenicity data be locally generated in populations where such heterologous vaccine is to be implemented. Therefore, this treatise advocates an experimental arm to generate robust evidence on immunogenicity, reactogenicity and safety of heterologous prime-booster within the African population, even in countries that have commenced the roll-out. This will provide an empirical evidence to guide this innovative approach aimed at ensuring equity and access to COVID-19 vaccines in LMICs, particularly countries within the African region.
